# The Louisville Medical College

**Published:** 1893-06

**Authors:** 


THE LOUISVILLE MEDICAL COLLEGE.—This institution is to be congratulated upon its elegant new building, just completed. The length of the building is 184 feet, and the width 87 feet; four stories high, with basement under the entire structure. It is built of stone, and the architecture is "modernized Romanesque," much taste and judgment being displayed in the finish and ornamentation. The college is situated on the northwest corner of First and Chestnut streets, and towering above all adjoining buildings, it is a conspicuous and graceful ornament to that aristocratic neighborhood. Heat and water are directed and controlled throughout the entire building, and every convenience and requirement is amply met.

The Louisville Medical College management deserve great credit for the zeal, energy and intelligent enterprise they have displayed even under past disadvantages. They have now overcome all obstacles and can rejoice. The institution is, and has always been a great favorite with the Texas boys, even when the old square antiquated stucco building was the best they could do, and Texans have contributed no little to the triumph of the school. The Secretary, Dr. Geo. M. Warner, says in a letter to the JOURNAL, "Texas was splendidly represented last session, (as it always is) and I would be at a loss to say which one of our many good students from the Lone Star State would not be worthy of special mention. They always rank high." We give in this issue a cut of the new building and publish the announcement of the next session. In writing to Dr. Warner for catalogue please mention this notice.

